# Instigation of NLRP3 inflammasome activation and glomerular injury in mice on the high fat diet: role of acid sphingomyelinase gene

**DOI:** 10.18632/oncotarget.8023

**Published:** 2016-03-10

**Authors:** Krishna M. Boini, Min Xia, Saisudha Koka, Todd W. Gehr, Pin-Lan Li

**Affiliations:** ^1^ Department of Pharmacology and Toxicology, School of Medicine, Virginia Commonwealth University, Richmond VA 23298, USA; ^2^ Division of Nephrology, School of Medicine, Virginia Commonwealth University, Richmond VA 23298, USA

**Keywords:** obesity, inflammasomes, ceramide, glomerulosclerosis, end-stage renal disease

## Abstract

Ceramide has been reported to initiate inflammasome formation and activation in obesity and different pathological conditions. The present study was performed to explore the role of acid sphingomyelinase (Asm) in the development of high fat diet (HFD)-induced inflammasome and activation and consequent glomerular injury. Asm knockout (*Asm^−/−^*) and wild type (*Asm^+/+^*) mice with or without Asm short hairpin RNA (shRNA) transfection were fed a HFD or normal chow for 12 weeks to produce obesity and associated glomerular injury. HFD significantly enhanced the Asm activity, ceramide production, colocalization of Nlrp3 (Nod-like receptor protein 3) with ASC (apoptosis-associated speck-like protein) or Caspase-1, NADPH-dependent superoxide (O_2_^•−^) production in glomeruli of *Asm^+/+^*mice than in control diet-fed mice. However, such HFD-induced increases in Asm activity, ceramide production, colocalization of Nlrp3 with ASC or Caspase-1, superoxide (O_2_^•−^) production was attenuated in *Asm^−/−^* or Asm shRNA-transfected wild-type mice. In consistency with decreased inflammasome formation, the caspase-1 activity and IL-1β production was significantly attenuated in *Asm^−/−^* or Asm shRNA-transfected wild-type mice fed a HFD. Morphological examinations showed that HFD-induced profound injury in glomeruli of *Asm^+/+^* mice which was markedly attenuated in A*sm^−/−^* mice. The decreased glomerular damage index in A*sm^−/−^* mice was accompanied by attenuated proteinuria. Fluorescent immunohistochemical examinations using podocin as a podocyte marker showed that inflammasome formation induced by the HFD were mostly located in podocytes as demonstrated by co-localization of podocin with Nlrp3. In conclusion, these observations disclose a pivotal role of Asm in the HFD-induced inflammasome formation and consequent glomerular inflammation and injury.

## INTRODUCTION

The prevalence of obesity is increasing worldwide and contributes to many health problems, including chronic kidney disease (CKD). CKD is now considered as one of the strongest risk factors for the morbidity and mortality in obese patients [1, 2]. Earlier studies reveal that adipose tissue, especially visceral fat generates bioactive substances that contribute to the pathophysiologic renal hemodynamic and structural changes leading to obesity-associated glomerular injury [3]. Mechanistically, previous studies have shown that obesity-induced glomerular sclerosis and ultimate end stage renal disease involved in chronic inflammation, abnormal vascular remodeling, rise in renal plasma flow, hyperfiltration and renal lipotoxicity [4]. Most recently, we have shown that formation and activation of Nlrp3 inflammasomes is an important initiating mechanism responsible for glomerular inflammation and injury in obese mice [5]. However, it remains unknown how the Nlrp3 inflammasomes is activated and thereby results in glomerular injury during obesity.

Recently sphingolipids have been recognized as signaling molecules involved in number of important cellular functions [6]. Sphingolipid, ceramide has been reported to initiate Nlrp3 inflammasome formation and activation in different pathological conditions including insulin resistance, obesity, Alzheimer's disease, cystic fibrosis and acute lung injury [6, 7]. Ceramide, released from the hydrolysis of membrane sphingomyelin by various sphingomyelinases such as acid sphingomyelinase (Asm) or neutral sphingomyelinase (NSM) or by *de novo* synthesis via serine palmitoyltransferase (SPT) and ceramide synthase [8, 9]. Ceramide is subsequently metabolized into sphingosine by ceramidases, and sphingosine can be further converted to S1P via sphingosine kinase in response to a variety of stimuli including proinflammatory cytokines, oxidative stress, and increased levels of free fatty acids. It was reported that Nlrp3 shRNA abolished the ceramide-induced inflammasomes formation, activation, proinflammatory cytokines and alveolar permeability in alveolar type II cells [6]. Furthermore, the acid sphingomyelinase heterozygous mice normalize the pulmonary ceramide levels and prevented the Nlrp3 inflammasome formation and activation in cystic fibrotic lungs [10]. However, it remains unknown whether Asm gene expression and regulation are implicated in the development of high fat diet (HFD)-induced Nlrp3 inflammasome formation and activation and consequent glomerular injury.

The present study was designed to investigate the role of acid sphingomyelinase (Asm) gene during obesity and explore its potential effects on Nlrp3 inflammasome activation and consequent glomerular sclerosis/injury. To test this hypothesis, we first performed a series of experiments using Asm^−/−^ and their wild type littermates on the normal chow or high fat diet to determine whether lack of Asm gene alters renal ceramide production, glomerular inflammasome activation and glomerular injury in mice during obesity. Then, we locally silenced renal Asm gene using shRNA and observed the effects of renal Asm deficiency on obesity-induced glomerular inflammasome formation, activation and corresponding injury. Our results demonstrate that Asm gene deficiency in the kidney attenuates the obesity-induced Nlrp3 inflammasome formation, activation and glomerular injury, ultimately preventing glomerulosclerosis.

## RESULTS

### Mice lacking Asm gene attenuates the HFD-induced Asm activity, ceramide production and Nlrp3 inflammasome formation

**Figure 1:**
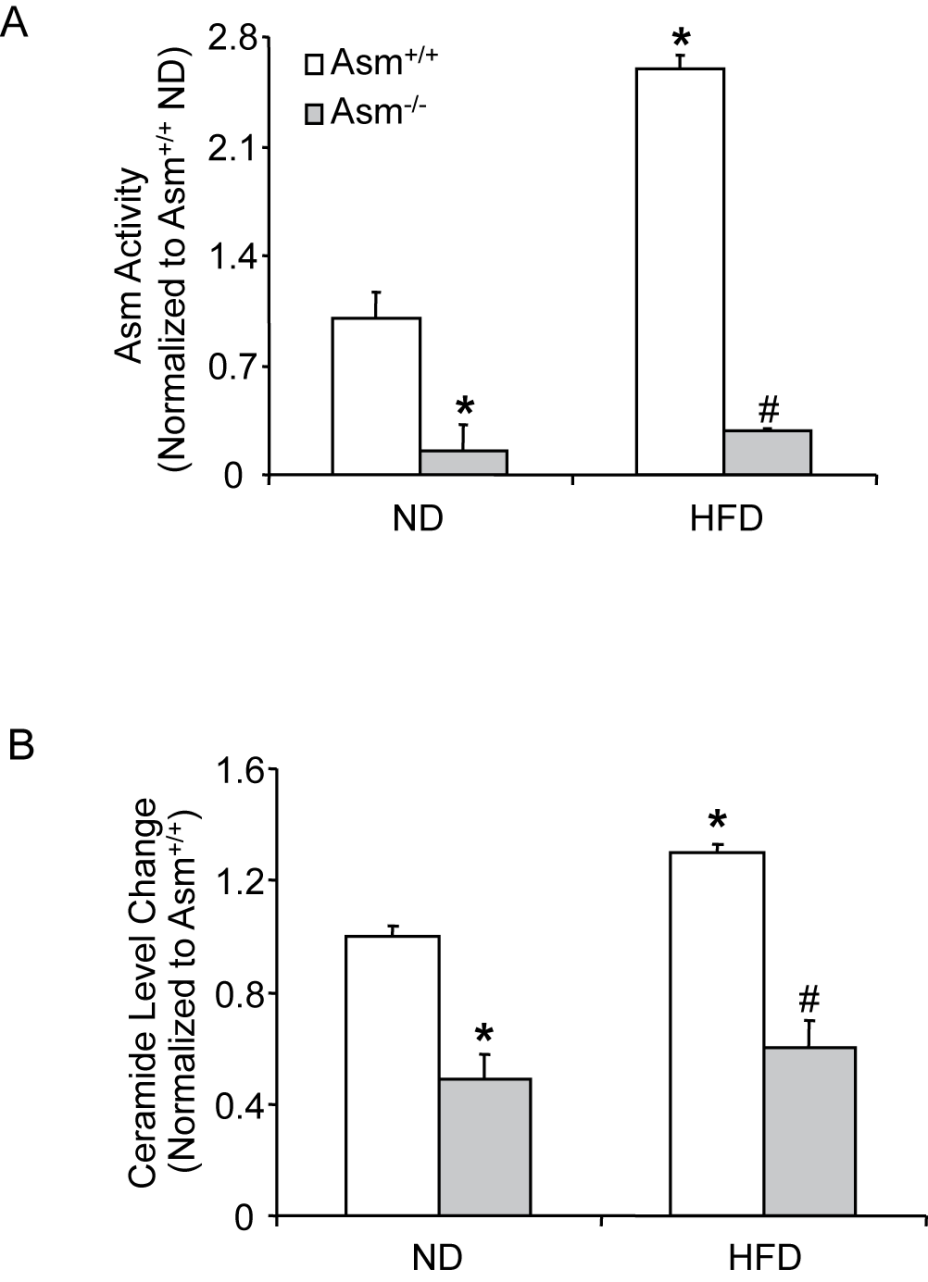
Effects of the normal diet and high fat diet on renal tissue Asm activity and total ceramide production in Asm^+/+^ and Asm^−/−^ mice Values are arithmetic means ± SE (n=6 each group) of Asm activity **A.,** total ceramide production **B.** in Asm^+/+^ and Asm^−/−^ mice with or without HF diet. * Significant difference (*P*<0.05) compared to the values from Asm^+/+^ mice fed on normal diet, ^#^ Significant difference (*P*<0.05) compared to the values from mice receiving the high fat diet. ND: Normal diet, HFD: High fat diet.

As shown in Figure 1A, the Asm activity was significantly lower in glomeruli of Asm^−/−^ than in Asm^+/+^ mice fed on normal diet. HFD treatment significantly increased the Asm activity in Asm^+/+^ mice when compared to normal diet fed mice. However this HFD- induced Asm activity was significantly attenuated in Asm^−/−^ mice (Figure 1A). Correspondingly, the total renal ceramide levels were lower in Asm^−/−^ than in Asm^+/+^ mice fed a normal diet. HFD significantly increased the glomerular total ceramide levels in Asm^+/+^ mice but not in Asm^−/−^ mice (Figure 1B).

**Figure 2:**
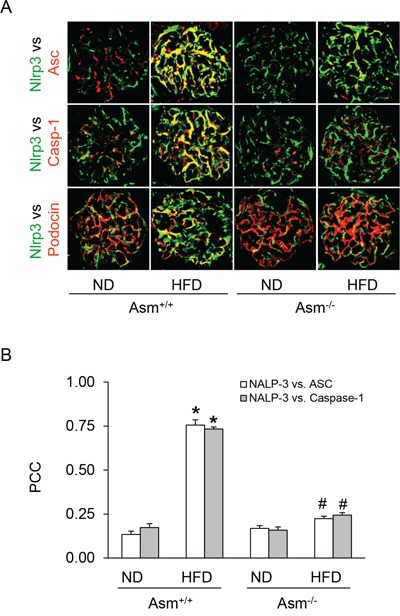
Attenuation of HFD-induced inflammasome formation in glomeruli of Asm^+/+^ and Asm^−/−^ mice fed a normal diet or high fat diet **A.** Colocalization of Nlrp3 (green) with ASC (red), Nlrp3 (green) with caspase-1 (red) and Nlrp3 (green) with podocin (red) in mouse glomeruli. **B.** Summarized data shows the fold changes of pearson coefficient correlation (PCC) for the colocalization of Nlrp3 (also known as Nalp3) with ASC and Nlrp3 with caspase-1 in glomeruli of Asm^+/+^ and Asm^−/−^ mice fed with ND or HFD. * Significant difference (*P*<0.05) compared to the values from control Asm^+/+^ mice, ^#^ Significant difference (*P*<0.05) compared to the values from Asm^+/+^ mice receiving the HFD. ND: Normal diet, HFD: High fat diet.

**Figure 3:**
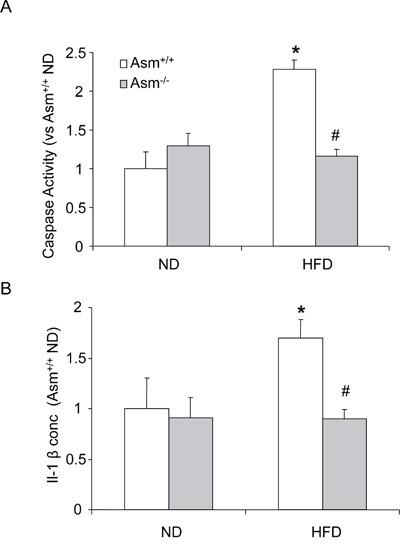
Effects of the normal diet and high fat diet on caspase-1 activity and IL-1β production in Asm^+/+^ and Asm^−/−^ mice Values are arithmetic means ± SEM (n=6 each group) of caspase-1 activity **A.,** IL-1B production **B.** in glomeruli of Asm^+/+^ and Asm^−/−^ mice fed with ND or HFD. Significant difference (*P*<0.05) compared to the values from control Asm^+/+^ mice, ^#^ Significant difference (*P*<0.05) compared to the values from Asm^+/+^ mice receiving the HFD. ND: Normal diet, HFD: High fat diet.

Next, we tested whether ASMase mediates the HFD-induced Nlrp3 inflammasome formation and activation in glomeruli of mice. Using confocal microscopy, we demonstrated that HFD increased the co-localization of Nlrp3 with ASC or Nlrp3 with caspase-1 in glomeruli of mice compared to normal diet fed mice. However, Asm^−/−^ mice attenuated the HFD-induced co-localization of Nlrp3 with ASC or Nlrp3 with caspase-1 in glomeruli of mice (Figure 2A). Furthermore, co-localization of Nlrp3 with podocin (podocyte marker) indicates enrichment of Nlrp3 inflammasomes in podocytes. The summarized data of quantitative co-localization of Nlrp3 with Asc or Nlrp3 with caspase-1 in glomeruli of mice were shown in (Fig. 2B. Biochemical analysis showed that HFD significantly increased the caspase activity and IL-1 β production in glomeruli of Asm^+/+^ mice fed a normal diet. However, Asm^−/−^ mice attenuated the HFD-induced caspase-1 activity and IL-1β production (Figure 3). Taken together, these results suggest that ASMase mediates the HFD-induced the NLRP3 inflammasome formation and activation in glomeruli of mice.

### Improvement of HFD-induced glomerular injury in mice lacking Asm gene

**Figure 4:**
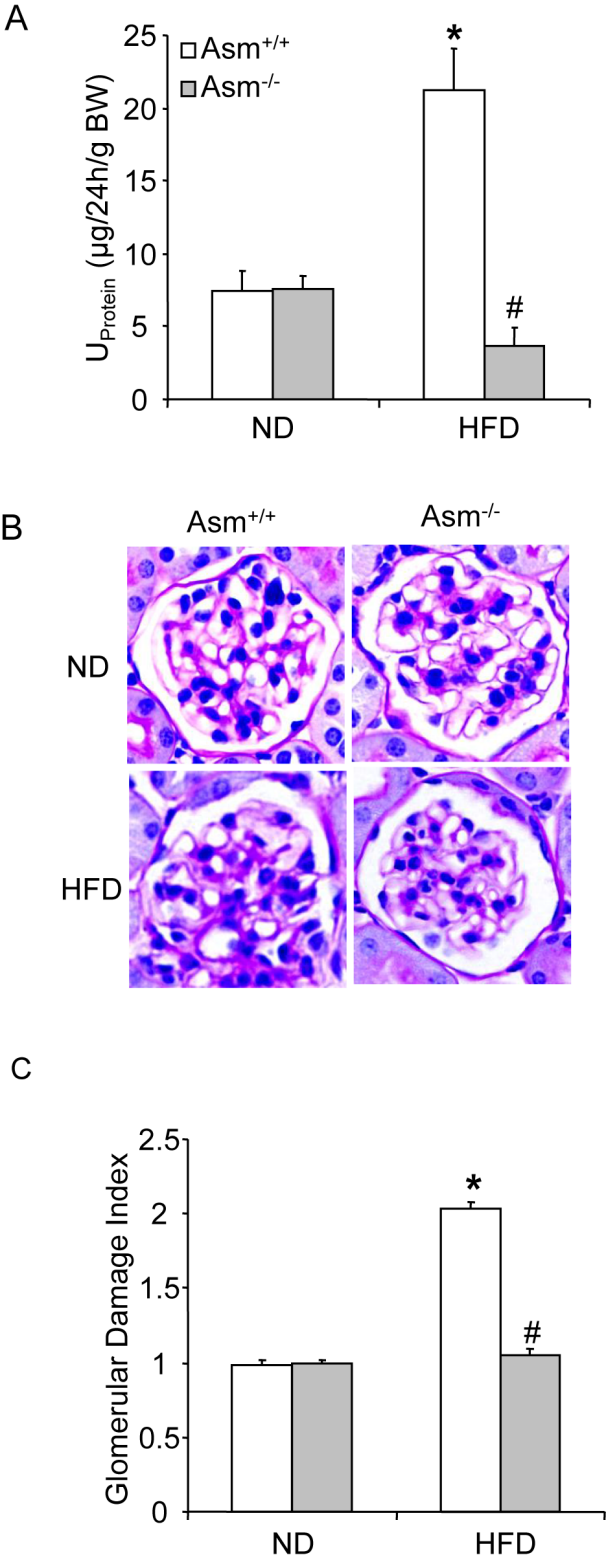
Effects of the normal diet and high fat Diet on glomerular injury in Asm^+/+^ and Asm^−/−^ mice Values are arithmetic means ± SEM (n=6 each group) of urinary protein excretion **A.** in Asm^+/+^ and Asm^−/−^ mice with or without the HFD. **B.** Photomicrographs show typical glomerular structure (original magnification, x400) in Asm^+/+^ and Asm^−/−^ mice fed with or without HFD **C.** Summarized data of glomerular damage index (GDI) by semi-quantitation of scores in 4 different groups of mice (n=6 each group). For each kidney section, 50 glomeruli were randomly chosen for the calculation of GDI. * Significant difference (*P*<0.05) compared to the values from Asm^+/+^ mice on the normal diet, ^#^ Significant difference (*P*<0.05) compared to the values from mice on the HFD.

As shown in Figure 4A, urinary protein excretion was similar in Asm^+/+^ and Asm^−/−^ mice fed a normal diet. However, HFD significantly increased the urinary total protein excretion in Asm^+/+^ mice, but not in Asm^−/−^ mice. By PAS staining, we observed a typical pathological change in glomerular sclerotic damage in Asm^+/+^ mice on the high fat diet such as glomerular capillary collapse and mesangial expansion. This pathology was not observed in Asm^−/−^mice. The glomerular damage index (GDI) was significantly higher in Asm^+/+^ mice fed a HFD compared to ND fed mice. However, the HFD-induced glomerular damage index was significantly attenuated in Asm^−/−^ mice (Fig. 4B and 4C).

### Blockade of local oxidative stress in the glomeruli of HFD fed mice lacking Asm gene

**Figure 5:**
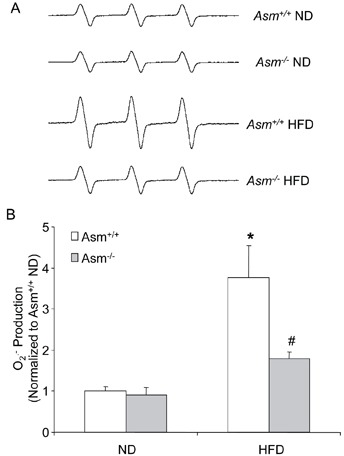
Effects of the normal diet and high fat diet on glomerular O_2_^•−^ production in Asm^+/+^ and Asm^−/−^ mice **A.** Representative ESR spectra traces for O_2_^•−^ production in Asm^+/+^ and Asm^−/−^mice. **B.** Values are arithmetic means ± SEM (n=5 each group) of O_2_^•−^ production in Asm^+/+^ and Asm^−/−^ mice fed with normal diet or high fat diet. * Significant difference (*P*<0.05) compared to the values from Asm^+/+^ mice on the normal diet, ^#^ Significant difference (*P*<0.05) compared to the values from mice on the high fat diet. ND: Normal diet, HFD: High fat diet.

As illustrated in Figure 5A, the ESR spectrometric curve exhibited significant increase in the amplitude of Nox-dependent O_2_^•−^ signals in the glomeruli of Asm^+/+^ mice on the high fat diet as compared with Asm^+/+^ mice on the normal diet. However, in HFD fed Asm^−/−^ mice failed to increase glomerular O_2_^•−^ production. These results were summarized in Figure 5B, showing that glomerular O_2_^•−^ production was similar in Asm^−/−^ and Asm^+/+^ mice on the normal diet, but increased by 3.5-fold in Asm^+/+^ mice fed a high fat diet. However, HFD-induced glomerular O_2_^•−^ production was much less in Asm^−/−^ mice compared with Asm^+/+^ mice.

### Efficiency of *in vivo* local transfection of Asm shRNA into the kidney

**Figure 6:**
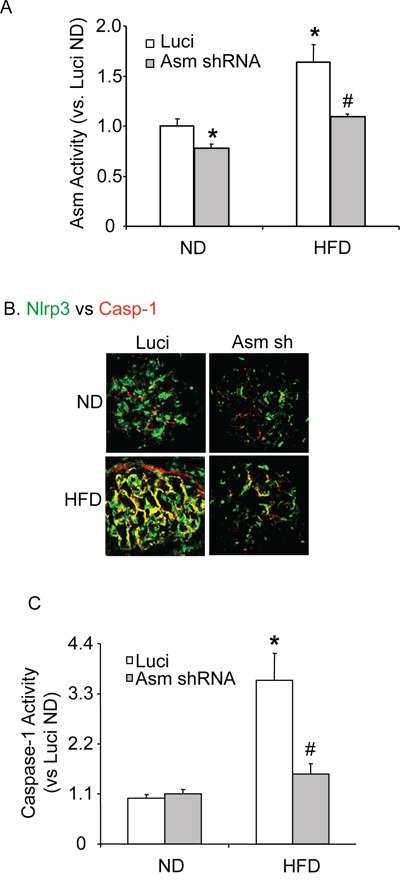
Renal Asm gene silencing efficiency and Nlrp3 inflammasome formation activation in C57BL/6J mice fed with or without high fat diet **A.** Values are arithmetic means ± SEM (n=4-6 each group) of Asm activity, **B.** Colocalization of Nlrp3 (green) with caspase-1 (red), **C.** caspase-1 activity in control or high fat diet-fed C57BL/6J mice with or without Asm shRNA transfection. * Significant difference (*P*<0.05) compared to the values from control mice fed on the normal diet, ^#^ Significant difference (*P*<0.05) compared to the values from mice on the high fat diet. ND: Normal diet, HFD: High fat diet.

We used an IVIS *in vivo* molecular imaging system to detect the expression of co-transfected luciferase gene, which insures an efficient delivery of target gene into the mouse kidney. The luciferase reporter gene was monitored in to the kidney of the living mouse after the injection of plasmid mixed with microbubbles under ultrasound force. Starting on day 3, the expression of luciferase gene persisted for 4 weeks. In the hemi-dissected kidney, all of the cortical regions were observed to exhibit efficient gene transfection (data was not shown). As illustrated in Figure 6A, Asm activity was significantly decreased in C57BL/6J WT mice transfected with Asm shRNA compared to control mice fed a normal diet. Compared to the normal diet, the HFD significantly increased Asm activity in glomeruli from mice receiving scrambled shRNA, but it had no effect on Asm activity in mice receiving Asm shRNA.

### Attenuation of HFD-induced inflammasome formation, glomerular injury and O_2_^•−^production by Asm gene silencing

**Figure 7:**
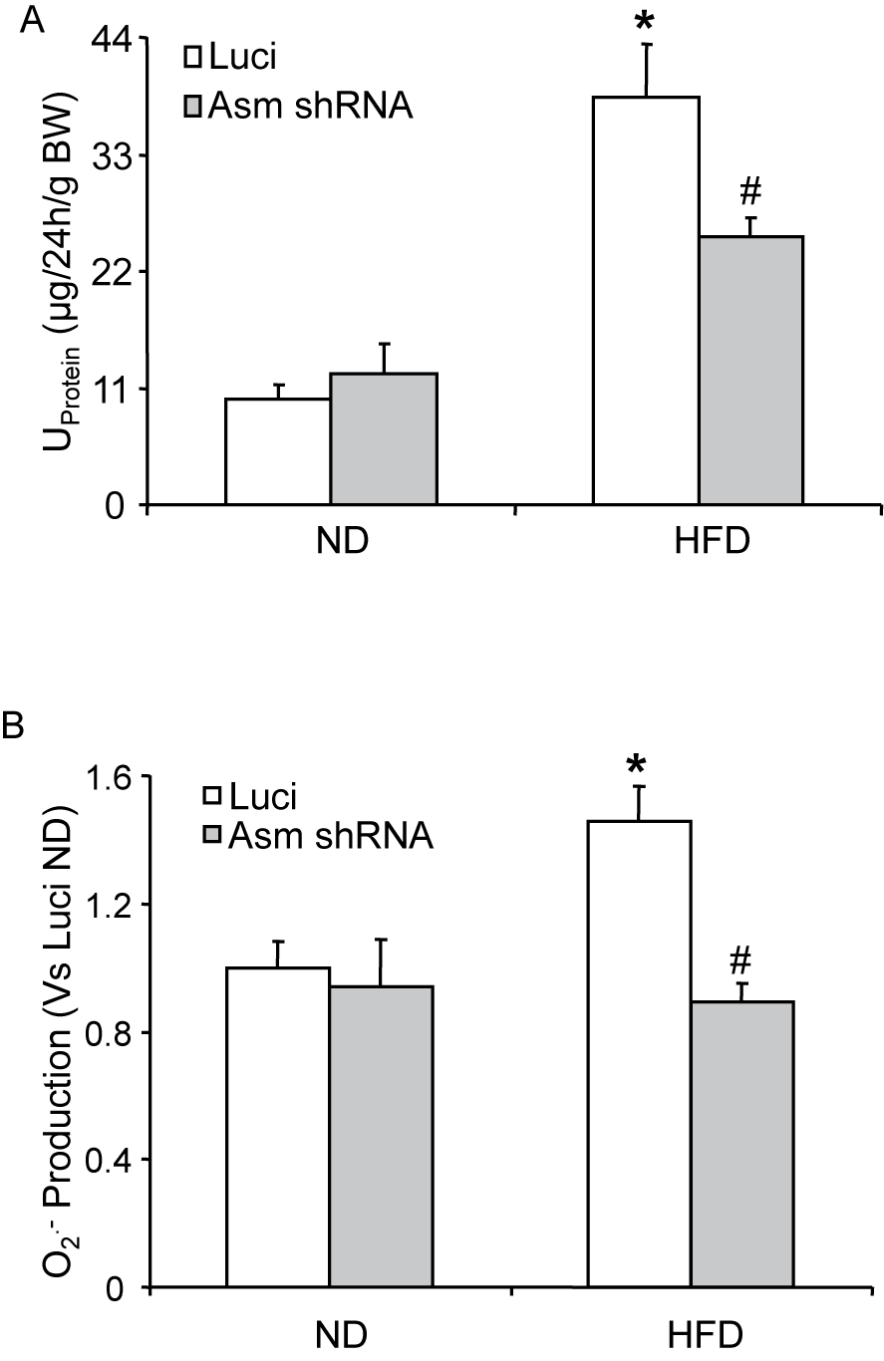
Effects of renal Asm gene silencing on glomerular injury and glomerular O_2_^•−^ production in C57BL/6J mice with or without the high fat diet Values are arithmetic means ± SEM (n=5-6 each group) of urinary total protein excretion A. and O_2_^•−^ production B. in C57BL/6J mice on the normal or HFD with or without Asm shRNA transfection. * Significant difference (*P*<0.05) compared to the values from control mice on the normal diet, ^#^ Significant difference (*P*<0.05) compared to the values from mice on the high fat diet. Luci: Scrambled shRNA, ND: Normal diet, HFD: High fat diet.

Further we determined whether Asm gene silencing locally in the kidney may attenuate the HFD-induced Nlrp3 inflammasome formation and protects against the glomerular injury. As shown in Figure 6B, HFD increased the co-localization of Nlrp3 with caspase-1 in glomeruli of scrambled shRNA transfected mice. However, such co-localization was not observed in glomeruli of Asm shRNA transfected mice, suggesting the attenuation of Nlrp3 inflammasome formation in glomeruli. In consistent with the decreased Nlrp3 inflammasome formation, caspase-1 activity was attenuated in Asm shRNA transfected mice (Figure 6C). The urinary protein excretion was similar in both scrambled and Asm shRNA transfected mice fed on normal diet. HFD diet treatment significantly increased the urinary total protein excretion when compared to the normal diet-fed mice. However, the Asm shRNA transfection significantly attenuated HFD-induced urinary total protein excretion (Figure 7A). Furthermore, the glomerular O_2_^•−^ production was similar in both scrambled and Asm shRNA transfected mice when they were on a normal diet. However, the HFD significantly increased the glomerular O_2_^•−^ production in scrambled shRNA transfected mice, but it had no effect on the glomerular O_2_^•−^production in mice with Asm shRNA transfection (Figure 7B).

## DISCUSSION

The present study was designed to explore the role of acid sphingomyelinase (Asm), a ceramide producing enzyme in obesity-induced NLRP3 inflammasome activation and associated glomerular injury. We found that HFD treatment enhanced the Asm activity and ceramide production, which was attributed to NLRP3 inflammasome activation in glomeruli and ultimately led to glomerulosclerosis. Inhibition of Asm gene prevented the obesity-induced inflammasome formation and subsequent glomerular injury. The findings for the first time demonstrate the critical role of Asm in the activation of Nlrp3 inflammasomes and subsequent glomerular dysfunction or sclerosis associated with obesity.

The inflammasomes as an intracellular machinery is responsible for the activation of inflammation in variety of tissues or organs [11]. Among different types of inflammasomes, the Nlrp3 inflammasome has been well characterized, which consists of a proteolytic complex formed by Nlrp3, the adaptor protein ASC, and caspase-1. Caspase-1 is activated when the inflammasome complex is formed to produce active IL-1β and IL-18 by cleavage of their precursors. Nlrp3 acts as the sensory component to recognize both endogenous and exogenous danger signals [7, 12–19], when ASC and caspase-1 are recruited to form a protein complex, where caspase-1 is activated [20–22]. The active caspase-1 not only proteolytically cleaves IL-1β and/or IL-18 into their biologically active form, but also produces other damaging molecules like damage-associated molecular patterns (DAMPs). Both may turn on inflammatory response and induce cell dysfunction or injury. NLRP3 inflammasome has been reported to be activated by bacterial toxins [12], ATP [19], monosodium urate crystals [14], β-amyloid [23], muramyldipeptide [13], cholesterol crystals [24] and other stimuli [25]. Functionally Nlrp3 inflammasome has been implicated in the pathogenesis of various metabolic diseases, including obesity, diabetes, gout, silicosis, and acetaminophen-induced liver toxicity [7, 14, 15, 25–29]. Most recently we have demonstrated that inhibition of NLRP3 inflammasomes either by apoptosis associated speck-like protein (ASC) gene knockout or ASC gene silencing attenuated the obesity-induced glomerular injury and podocyte injury [5]; however, it remains unknown how obesity-induces Nlrp3 inflammasome activation in glomeruli. Accumulating evidence demonstrated that sphingolipids, acid sphingomyelinase and its product ceramide has been reported to initiate inflammasome formation and activation in different pathological conditions. Knockout of the NLRP3 inflammasome (NLRP3^−/−^, ASC^−/−^, and caspase-1^−/−^) significantly protected mice from HFD-induced obesity, increased adiposity, insulin resistance, glucose intolerance, and inflammation [7, 16, 30]. The expression of the NLRP3 inflammasome subunits in adipose tissue correlates directly with body weight in mouse models and obese individuals with type 2 diabetes mellitus [7]. The mechanism of HFD-induced inflammasome activation may be due to the high production of the fatty acid metabolites ceramide and palmitate, as saturated fatty acids have been shown to induce inflammasome activation through a mechanism that involves defective autophagy and the accumulation of mtROS. In this regard, Vandanmagsar *et al*. showed that adding ceramide to adipose tissue explants led to NLRP3-dependent IL-1β production, suggesting that ceramide acts a danger signal to stimulate the NLRP3 inflammasome activation [7], however, there have been no studies to date exploring the effects of ceramide production on obesity-induced inflammasome activation in podocytes and associated glomerular injury. In the current study, we sought to determine whether ASMase mediates HFD-induced inflammasome formation in glomeruli and associated glomerular injury.

Using Asm^−/−^ mice and their wild type littermates, we first induced obesity by feeding them a high fat diet for 12 weeks. It was found that high fat diet treatment significantly increased the ceramide levels in glomeruli or Asm^+/+^ compared to normal diet fed mice, however Asm^−/−^ mice attenuated the HFD-induced ceramide production. Furthermore, Asm activity in renal tissues were significantly increased in high fat diet fed Asm^+/+^ mice, but not in Asm^−/−^ mice. These results together suggest that HFD-induced glomerular ceramide level mainly due to the activation of acid sphingomyelinase. Recent reports suggest that activated ceramide induces caspase-1 activation, IL-1β production in an Nlrp3 inflammasome dependent mechanism in obesity or acute lung injury [6, 7]. Indeed, our present study, demonstrated that increased ceramide production induced the formation of NLRP3 inflammasomes in glomeruli as shown by colocalization of NLRP3 with ASC or NLRP3 with caspase-1 in Asm^+/+^ mice. Using podocin as a podocyte marker, our confocal observations demonstrated that obesity-induced inflammasome formation in glomeruli was mostly located in podocytes as demonstrated by the colocalization of Nlrp3 with podocin. These colocalizations were substantially blocked in mice lacking Asm gene. Moreover, the biochemical analysis showed that obesity increased the caspase-1 activity and IL-1β production in Asm^+/+^ mice but not in Asm^−/−^ mice, suggesting the essential role of Asm gene in mediating the obesity-induced NLRP3 inflammasome formation and activation in glomeruli of mice.

Acid sphingomyelinase and its product ceramide has been implicated in the regulation of kidney function [8, 9, 31–33] in different pathological conditions such as obesity, hyperhomocysteinemia or diabetes. More recently, our group demonstrated that ceramide importantly contributes to the development of chronic glomerular injury associated with obesity or hyperhomocysteinemia and thereby ceramide may serve as an important mechanism of end-stage renal disease [8, 9]. Moreover, Nlrp3 inflammasome has been implicated in the regulation of kidney function [34–36]. Recent studies from our laboratory and others demonstrated the role of inflammasome in hHcys or obesity-induced glomerular injury [5, 17, 37], acute ischemia/reperfusion-induced kidney injury [35], unilateral ureteral obstruction [38, 39] and renal biopsies from patients with non-diabetic kidney disease [36]. Inhibition of inflammasomes attenuated the proteinuria and kidney function. In this study, we tested, whether obesity-induced glomerular injury through Nlrp3 inflammsome activation in Asm^+/+^ and knockout mice. The present study showed that in accordance with decreased inflammasome formation in HFD fed Asm^−/−^ mice, urinary protein excretion and glomerular injury/sclerosis were significantly blocked compared with Asm^+/+^ mice on the high fat diet, suggesting the contribution of ASMase to the inflammasome formation in podocytes and thereby leading to the obesity-induced glomerular injury. Therefore, this acid sphingomyelinase could be a target of therapeutic strategy for obesity-induced glomerular injury or sclerosis.

To further explore the mechanism of obesity-induced Nlrp3 inflammasome activation in HFD fed mice, we determined the NADPH oxidase derived O_2_^•−^ production in Asm^+/+^ and Asm^−/−^ mice. It is well documented that several mechanisms underlying inflammasome activation have been reported, including lysosome rupture, ion channel gating, and reactive oxygen species (ROS) activation [30, 37]. Activation of the NLRP3 inflammasome by increased ROS, the most widely accepted and considered to be the most plausible mechanism, suggests that this inflammasome is a general sensor for changes in cellular oxidative stress. Indeed, the present study showed that HFD significantly increased the NADPH oxidase-dependent O_2_^•−^ production in Asm^+/+^ mice but not in Asm^−/−^ mice. These results confirm the imperative role of Asm in mediating O_2_^•−^ production through the activation of NADPH oxidase in glomeruli during obesity.

To further confirm our results, we address the role of Asm gene in mediating obesity-induced inflammasome formation and consequent glomerular injury, a local gene silencing strategy was used in the present study, where an ultrasound microbubble-mediated plasmid delivery was employed to introduce Asm shRNA into the kidney. It was demonstrated that this method was highly efficient in delivering plasmids into renal cells *in vivo*, which led to gene transfection and expression in most renal cells (90%) as confirmed the earlier reports [5, 8, 17, 40]. By an *in vivo* molecular imaging system to daily monitor the efficiency of Asm gene transfection in the kidney in living animals, we showed that the transgene or shRNA expression vector (with luciferase gene as an indicator) could be detected even 3 days after gene transfection and lasted for 4 weeks observed (data was not shown). This *in vivo* transgene monitoring importantly guided our functional studies to define the role of Asm gene in mediating glomerular damage associated with obesity. After completion of functional protocols, Asm activity was analyzed to confirm the Asm gene silencing efficiency in shRNA transfected kidneys. In our present study, we found that glomerular ASM activity, co-localization of Nlrp3 with ASC, caspase-1 activity and glomerular O_2_^•−^ production was significantly decreased. It was also demonstrated that silencing Asm gene in the kidney ameliorates proteinuria. Taken together, these results from mice with local renal Asm gene silencing further support the conclusion above drawn from studies using Asm knockout mice that Asm inhibition or gene silencing abolishes obesity-induced inflammasome formation and thereby protect kidney from obesity-induced glomerular injury. In conclusion, the present study demonstrated that Asm plays a pivotal role in obesity-induced inflammasome formation, activation and consequent glomerular injury. Therefore, targeting Asm may be an important therapeutic strategy to prevent inflammasome activation and thereby protect glomeruli from obesity-induced injury.

## MATERIALS AND METHODS

### Animals

Eight weeks old male *Asm*^−/−^ mice and their wild type littermates were used in the present study [8, 41]. The mice were fed either a low fat diet (LFD: D 12450B, 10 kcal % fat, Research Diets, New Brunswick, NJ) or a high fat diet (HFD: D 12492, 60 kcal % fat, Research Diets, New Brunswick, NJ) for 12 weeks [5, 9]. In another series, eight weeks old male C57BL/6J wild type mice (Jackson Laboratories, Bar Harbor, ME), Asm shRNA or a scrambled shRNA (Origene, Rockville, MD, USA) plasmid with a luciferase expression vector was co-transfected into the kidneys via intrarenal artery injection using the ultrasound microbubble system as we described previously [8]. After the delivery of plasmids into the kidney, mice were fed either a normal diet or HFD for 12 weeks. All protocols were approved by the Institutional Animal Care and Use Committee of the Virginia Commonwealth University.

### Gene transfer into the kidney by ultrasound-microbubble technique

The procedures for the ultrasound-microbubble gene transfer technique include: (1) mixing luciferase and designated plasmid (50 μg) in saline and microbubble (Optison, GE HealthCare) at a ratio of 3:1 vol/vol in 0.3 ml and injecting the mixed solution by 30G needle into the left renal artery with temporary clipping of the renal artery and vein (<5 min); (2) applying the ultrasound transducer (Ultax UX-301; Celcom Medico Inc., Japan) directly onto one side of the left kidney with a continuous-wave output of 1 MHz ultrasound at 10% power output, for a total of 60 s at 30-s intervals; (3) Finally, the renal artery and vein were unclipped after needle was taken off, and renal blood flow recovered. At the same time, one or two cotton tips were used to prevent bleeding at the puncture point of the renal artery [5, 8, 17, 33, 40].

### Acid sphingomyelinase (ASMase) activity

The activity of ASMase was determined as we described previously [2, 8, 42]. Briefly, *N*-methyl-[^14^C]-sphingomyelin was incubated with renal cortex tissue homogenates, and the metabolites of sphingomyelin, [^14^C]-choline phosphate was quantified. An aliquot of homogenates (20 μg) was mixed with 0.02 μCi of *N*-methyl ^14^C-sphingomyelin in 100 μl acidic reaction buffer containing 100 mmol/L sodium acetate, and 0.1% Triton X-100, pH 5.0, and incubated at 37°C for 15 min. The reaction was terminated by adding 1.5 ml chloroform:methanol (2:1) and 0.2 ml double-distilled water. The samples were then vortexed and centrifuged at 1,000 *g* for 5 min to separate into two phases. A portion of the upper aqueous phase containing ^14^C-choline phosphate was transferred to scintillation vials and counted in a Beckman liquid scintillation counter. The choline phosphate formation rate (nmol•min^−1^•mg protein^−1^) was calculated to represent the enzyme activity.

### Liquid chromatography–electrospray ionization tandem mass spectrometry (LC-ESI-MSMS) for quantitation of ceramide

Separation, identification and quantitation of ceramide in plasma were performed by LC/MS [2, 8]. The HPLC equipped with a binary pump, a vacuum degasser, a thermostated column compartment and an autosampler (Waters, Milford, MA, USA). The HPLC separations were performed at 70°C on a RP C18 Nucleosil AB column (5 μm, 70 mm × 2 mm i.d.) from Macherey Nagel (Düren, Germany). The mobile phase was a gradient mixture formed as described [43]. The plasma lipids were extracted according to previous studies. To avoid any loss of lipids, the whole procedure was performed in siliconized glassware. MS detection was carried out using a Quattro II quadrupole mass spectrometer (Micromass, Altrincham, England) operating under MassLynx 3.5 and configured with a Z-spray electrospray ionization source. Source conditions were described as previously [43].

### Confocal microscopic detection of inflammasome protein complexes

Indirect immunofluorescent staining was used to determine colocalization of the inflammasome proteins in glomeruli of the mouse kidney, which indicate the formation of inflammasome molecular complex. Frozen kidney tissue slides were fixed in acetone and then incubated overnight at 4°C with either goat anti- Nlrp3 (1:100, Novus Biologicals) and rabbit anti-Asc (1:50, Enzo Lifesciences), or goat anti- Nlrp3 (1:200) and mouse anti-caspase-1 (1:100, Santa Cruz Biotechnology). To further confirm the presence of the inflammasomes specifically in podocytes of the mouse glomeruli, Nlrp3 or caspase-1 was co incubated with a podocin antibody (1:400; Sigma, St. Louis, MO). Double immunofluorescent staining was achieved by incubating with either Alexa-488 or Alexa-555-labeled secondary antibodies for 1 hour at room temperature. After washing, slides were mounted with a DAPI-containing mounting solution, and then observed with a confocal laser scanning microscope (Fluoview FV1000, Olympus, Japan). As previously described [17, 40], images were analyzed by the Image Pro Plus 6.0 software (Media Cybernetics, Bethesda, MD), where colocalization was measured and expressed as the Pearson Correlation Coefficient (PCC).

### Caspase-1 activity, IL-1β production

Caspase-1 activity in glomeruli was measured by a commercially available colorimetric assay kit (Biovision, Mountain View, CA). IL-1β production in glomeruli was measured by a commercially available ELISA kit (R&D System, Minneapolis, MN), according to the manufacturer's instructions [17, 37].

### Morphological examinations

The fixed kidneys were paraffin-embedded, and sections were prepared and stained with Periodic acid–Schiff stain. Glomerular damage index (GDI) was calculated from 0 to 4 on the basis of the degree of glomerulosclerosis and mesangial matrix expansion as described previously [9, 40]. In general, we counted 50 glomeruli in total in each kidney slice under microscope, when each glomerulus was graded level 0-4 damages. 0 represents no lesion, 1+ represents sclerosis of <25% of the glomerulus, while 2+, 3+, and 4+ represent sclerosis of 25% to 50%, >50% to 75%, and >75% of the glomerulus. A whole kidney average sclerosis index was obtained by averaging scores from counted glomeruli. This observation was examined by two independent investigators who were blinded to the treatment of the experimental groups [8, 9, 17, 37, 40].

### Urinary total protein excretion measurement

The 24-hour urine samples were collected using metabolic cages and subjected to total protein excretion measurements respectively [8, 9, 40]. Total protein content in the urine was detected by Bradford method using a UV spectrophotometer.

### Delivery of Asm shRNA into the kidneys by ultrasound-microbubble technique

Asm shRNA or a scrambled shRNA plasmid with a luciferase expression vector was used to co-transfect the kidneys via intrarenal artery injection using the ultrasound-microbubble system. These experiments were performed to test whether local silencing Asc gene expression in podocytes alters obesity-induced glomerular injury. A full description of the procedures for the ultrasound-microbubble gene transfer technique can be found in our previous studies [8, 17, 40]. To monitor the efficiency of gene expression through somatic plasmid transfection daily, mice were anesthetized with isoflurane, and an aqueous solution of luciferin (150 mg/kg) was injected intraperitoneally 5 minutes before imaging. The anesthetized mice were imaged using the IVIS200 *in vivo* molecular imaging system (Xenogen, Hopkinton, MA, USA). Photons emitted from luciferase-expressing cells within the animal body and transmitted through tissue layers were quantified over a defined period of time ranging up to 5 minutes using the software program Living Image as program (Xenogen). The inhibitory efficiency of gene expression by Asm shRNA was further confirmed by detection of Asc level in mouse renal cortex using real-time RT-PCR.

### Electronic spin resonance (ESR) analysis of O_2_^•−^ production

For detection of Nox-dependent O2 ^•−^ production [2, 8], proteins from mouse renal cortex were extracted using sucrose buffer and resuspended with modified Kreb's–Hepes buffer containing deferoximine (100 mM, Sigma) and diethyldithiocarbamate (5 mM, Sigma). The Nox-dependent O2 .- production was examined by addition of 1mM NADPH as a substrate in 50 mg protein and incubated for 15 min at 37^°^C in the presence or absence of SOD (200 U/ml), and then supplied with 1mM O2 ^•−^ specific spin trap 1-hydroxy-3-methoxycarbonyl-2,2,5,5-tetramethylpyrrolidine (CMH, Noxygen, Elzach, Germany). The mixture was loaded in glass capillaries and immediately analyzed for O2 ^•−^ production kinetically for 10 min in a Miniscope MS200 electromagnetic spin resonance (ESR) spectrometer (Magnettech Ltd, Berlin, Germany). The ESR settings were as follows: biofield, 3350; field sweep, 60 G; microwave frequency, 9.78 GHz; microwave power, 20mW; modulation amplitude, 3 G; 4,096 points of resolution; receiver gain, 20 for tissue and 50 for cells. The results were expressed as the fold changes of control.

### Statistical analysis

Data are provided as arithmetic means ± SEM; *n* represents the number of animals in each experiment. All data were tested for significance using ANOVA or paired and unpaired Student's t-test as applicable. The glomerular damage index was analysed using a nonparametric Mann-Whitney rank sum test. Only results with p<0.05 were considered statistically significant.
